# Keep in Touch (KIT): feasibility of using internet-based communication and information technology in palliative care

**DOI:** 10.1186/s12904-017-0203-2

**Published:** 2017-05-06

**Authors:** Qiaohong Guo, Beverley Cann, Susan McClement, Genevieve Thompson, Harvey Max Chochinov

**Affiliations:** 1 Manitoba Palliative Care Research Unit, CancerCare Manitoba, 3017-675 McDermot Ave, Winnipeg, MB R3E 0V9 Canada; 2 Department of Psychiatry, University of Manitoba, Winnipeg, Canada; 3 College of Nursing, University of Manitoba, Winnipeg, Canada

**Keywords:** Communication and information technology, Keep in touch, Palliative care, Palliative inpatients, Family members

## Abstract

**Background:**

Confinement to an in-patient hospital ward impairs patients’ sense of social support and connectedness. Providing the means, through communication technology, for patients to maintain contact with friends and family can potentially improve well-being at the end of life by minimizing social isolation and facilitating social connection. This study aimed to explore the feasibility of introducing internet-based communication and information technologies for in-patients and their families and to describe their experience in using this technology.

**Methods:**

A cross-sectional survey design was used to describe patient and family member experiences in using internet-based communication technology and health care provider views of using such technology in palliative care. Participants included 13 palliative in-patients, 38 family members, and 14 health care providers. An iPad or a laptop computer with password-protected internet access was loaned to each patient and family member for about two weeks or they used their own electronic devices for the duration of the patient’s stay. Quantitative and qualitative data were collected from patients, families, and health care providers to discern how patients and families used the technology, its ease of use and its impact. Descriptive statistics and paired sample t-tests were used to analyze quantitative data; qualitative data were analyzed using constant comparative techniques.

**Results:**

Palliative patients and family members used the technology to keep in touch with family and friends, entertain themselves, look up information, or accomplish tasks. Most participants found the technology easy to use and reported that it helped them feel better overall, connected to others and calm. The availability of competent, respectful, and caring technical support personnel was highly valued by patients and families. Health care providers identified that computer technology helped patients and families keep others informed about the patient’s condition, enabled sharing of important decisions and facilitated access to the outside world.

**Conclusions:**

This study confirmed the feasibility of offering internet-based communication and information technologies on palliative care in-patient units. Patients and families need to be provided appropriate technical support to ensure that the technology is used optimally to help them accomplish their goals.

## Background

High-quality palliative care includes attending to a patient’s important relationships [1]. The support of loved ones helps buffer some of the effects of a terminal illness [2], minimizes despair and improves psycho-spiritual well-being [3, 4]. Maintaining relationships and open communication with family and friends can bolster patients’ sense of well-being [5]. Confinement to an in-patient hospital ward can impair a person’s sense of social support and connectedness, especially when loved ones are far away or mobility or economics prevents visiting. Providing the means, through communication technology, for patients to maintain contact with friends and family can potentially improve well-being at the end of life by minimizing social isolation and facilitating social connection [6].

The use of technology is increasingly common in hospital settings. Beyond the more familiar telemedicine tools for professional-patient communication, other computer-mediated technologies have been evaluated for their ability to reduce social isolation, enhance relationships, or support people facing illness or disability [7, 8]. iPads or tablet computers are being provided in hospitals to help patients access cancer treatment and support services, assist them in communicating with staff using video-calling software, connect with family and friends outside of the hospital, and access entertainment [9, 10]. Research indicates that the majority of patients enjoy using the iPad, helping them endure treatments and providing a form of relaxation and distraction [10].

Communication and information technologies have been used in palliative care, long-term care, and rural care facilities to facilitate physician-to-patient and patient-to-family communication [1, 11–18], offer patient education [19], allow patients to access entertainment such as visits to virtual museums [18] and attend special events such as weddings [1]. Case reports on use of internet-based communication technologies in palliative and hospice care for patient-family communication and social networks, though limited, suggest that these technologies relieve patients’ physical and spiritual suffering [20, 21], help patients stay in contact with family and friends who live far away and strengthen relationships with loved ones [18], help bring closure to families, promote healthy grieving and help the dying patient reconnect and reconcile with family members [1, 20].

Between 2011 and 2013, we conducted a two-phase project entitled Keep in Touch (KIT). In the first phase, a diverse group of key informants, including patients, families, information technology (IT) experts, and direct care providers, were consulted regarding their perspectives on introducing internet-based communication and information technologies at the bedside on in-patient palliative care units. Results affirmed the acceptability of offering these technologies, providing the foundation for trialing these technologies on a palliative in-patient unit [22]. Phase 2 of the KIT project explores the feasibility of offering internet-based communication and information technologies for palliative in-patients, and describes patient, family and health care provider experiences in using these technologies.

## Method

We conducted a feasibility study of internet-based communication and information technology, engaging a convenience sample of patients and family members recruited from a 30-bed palliative care unit. This unit was outfitted with a suite of internet-based (hard-wired or wireless) communication and information tools. Patients were eligible if they were in-patients on the participating palliative care unit, cognitively intact (based on clinical consensus) and able to understand and speak English. Family members or friends were eligible if they were visiting an in-patient on the participating unit and able to understand and speak English. Family participation was independent of whether the patients were too ill or not interested in the study. Eligible in-patients and family members were identified by unit staff, who asked for permission to have the research staff contact the patient/family member to provide more information about the study. In addition, patients/family members who detected the study’s signal and enquired about the availability of Wi-Fi were provided with contact information for the research staff. Participants thus recruited were considered “self-referred”. Health care providers (HCPs) were eligible for the study if they were employed on this palliative care unit and were actively involved in the care of patient or family member participants. They were recruited by email invitation sent by the unit manager and a poster in the staff conference room. The study was approved by the University of Manitoba Research Ethics Board and was approved by the hospital for site access.

### Technology set-up and facilitation

One consumer Digital Subscriber Line (DSL) service from the local phone company was used and connected to a consumer router. One consumer Wi-Fi hotspot was installed above the ceiling tiles in each of the two sides of the palliative care unit. The speed of the internet connection averaged 5 Mbps download and 0.75 Mbps upload. This provided round-the-clock Wi-Fi internet access not previously available in patient rooms. A remote monitor was installed to track all Wi-Fi internet traffic on the unit for the duration of the study. Wi-Fi internet access was password-protected to ensure that only people agreeing to take part in the study would be able to access it, and to prevent possible overloading of the temporarily-supplied bandwidth and “contamination” of the data being collected on the remote monitor.

Patients and family members were offered the use of either an Apple 16GB iPad or a Lenovo ThinkPad laptop with internet access for a maximum of two weeks during which time they were asked to keep a log of at least five days’ usage. Some participants chose to use their own devices. A research assistant was available on a daily basis to help participants use the technology to meet their goals. Through frequent check-ins, and on an on-call basis (Monday to Friday day shift, including the occasional weekend), the research assistant was available to guide participants in the use of the technology and to trouble shoot problems as they arose.

### Data collection

At the beginning of the study, each patient and/or family member participant was asked to provide basic demographic information, basic health information (patient only), and information about their general interests and experience with computers; to outline any current problems or challenges with maintaining social contacts; and their expectations of the communication and information technology. Patients completed a series of measures including: 1) the Blessed Orientation-Memory-Concentration (BOMC) test [23], a six-item screening test of cognition. Scores from each of the six items are multiplied to yield a weighted score, ranging from 0 to 28. People scoring 15 or greater were considered ineligible; 2) the Victoria Hospice Palliative Performance Scale (PPS) [24], a clinician-rated measure of patients’ performance status in 10% decrements from 100% (healthy) to 0% (death); 3) a self-rated quality of life (QOL) and satisfaction with QOL on a scale from 1 (poor/not satisfied at all) to 10 (excellent/very satisfied); 4) the UCLA Loneliness Scale [25], a 20-item scale to measure subjective feelings of loneliness and social isolation (the higher the score, the greater the feelings of loneliness); and 5) the Multidimensional Scale of Perceived Social Support (MSPSS) [26], a 12-item scale to measure perceived social support from family, friends, and significant others. The lowest overall score on the scale is 12 (least support) to 84 (most support).

During the hospital stay, patients and family members were asked to keep a daily hard-copy log of technology-related activities including: type and frequency of technology-facilitated contacts, means of contact, relationship of those contacted and their general location, and types of on-line information and entertainment sought. The hard-copy logs were supplemented by data gathered via the remote monitor. Upon completion of the study, participants were asked to provide summary data to evaluate their overall experience and satisfaction with using the technology. Family members of patients who died during the study were contacted several weeks to months following the deaths to evaluate their KIT study experience. In addition, the research assistant recorded the time required to orient and facilitate patients and family members with the technology and trouble-shoot any problems.

Health care providers were asked their observations of the utility of the technology for their patients. A convenience sample of direct care providers was surveyed in months 2, 4, 6, and 8 of the study, asking them for a summative evaluation of the perceived benefits or detriments of offering internet-based communication and information technology to patients and their families. Their demographic information was collected before the first survey.

### Data analysis

Demographic information and quantitative data were analyzed using SPSS 22.0. Descriptive statistics (frequency, means, and standard deviations) were used to analyze demographic information and quantitative data collected in surveys. Paired sample t-tests were employed to compare patients’ QOL, satisfaction with QOL and UCLA Loneliness scores before and after using the KIT technology. Qualitative data were subject to content analysis, and constant comparative techniques were used to identify themes, consensus, and major differences.

## Results

### Participants’ characteristics

Of 95 patients and family members who were referred to this study, 51 people (53.7%) took part, including13 patients and 38 family members. The most common reasons for declining were “not interested” and “inappropriate timing”. Of the 51 participants, 30 individuals were referred to the study by staff and 21 were self-referred; 39 individuals, either patient or family member, participated alone while 12 individuals participated in a dyad, meaning a patient and their family member, each contributed their own data. In several instances family members helped the patient complete the study logs and facilitated the use of the computer. Patient and family demographic information is shown in Table 1. The patient BOMC scores ranged from 0 to 8; the mean was M = 3.62 (SD = 2.82), indicating all patients were cognitively competent to participate. PPS Scores ranged from a minimum of 30 to a maximum of 60; the mean was M = 40.83 (SD = 9.00).

**Table 1:** Characteristics of patients (*N* = 13) and family members (*N* = 38)

Characteristics	Patients N (%)	Family members N (%)
Age (years)		
Mean (SD)	69.25 (10.83)	50.35 (13.32)
Range	42–82	25–81
Gender		
Female	8 (61.5)	23 (60.5)
Male	5 (38.5)	15 (39.5)
Education		
Completed post secondary	3 (23.1)	23 (60.5)
Some post secondary	5 (38.5)	11 (28.9)
Completed high school	3 (23.1)	2 (5.3)
Some high school/elementary	2 (15.4)	2 (5.3)
Marital Status		
Married	7 (53.8)	28 (73.7)
Widowed	2 (15.4)	2 (5.3)
Divorced	3 (23.1)	1 (2.6)
Never married	1 (7.7)	6 (15.8)
Other	0 (0.0)	1 (2.6)
Primary Diagnosis		
Cancer	10 (76.9)	
Other	3 (23.1)	
Relationship to Patient		
Spouse		7 (18.4)
Son or Daughter		22 (57.9)
Sibling		1 (2.6)
Friend		1 (2.6)
Other		3 (7.9)
Missing		4 (10.5)
Occupation		
Retired	10 (76.9)	10 (26.3)
Education, health care, social services	1 (7.7)	8 (21.1)
Other professional, technical or admin	1 (7.7)	7 (18.4)
Business owner/senior management	0 (0.0)	3 (7.9)
Sales and service	0 (0.0)	2 (5.3)
Trades	0 (0.0)	2 (5.3)
Leave/student	0 (0.0)	2 (5.3)
Homemaker	1 (7.7)	1 (2.6)
No response	0 (0.0)	3 (7.9)
Household		
< $20,000/year	2 (15.4)	0 (0.0)
$21,000 to $40,000/year	1 (7.7)	5 (13.2)
$41,000 to $60,000/year	1 (7.7)	3 (7.9)
$61,000 to $80,000/year	3 (23.1)	5 (13.2)
$81,000 to $100,000	0 (0.0)	4 (10.5)
> $100,000/year	1 (7.7)	10 (26.3)
Preferred not to answer	5 (38.5)	10 (26.3)
Missing	0 (0.0)	1 (7.7)

Fourteen HCPs on the palliative care unit participated in the study over its eight-month duration. Table 2 shows health care providers’ demographic characteristics. They contributed data at two-month intervals, so long as they had new observations to report. At two months, seven HCPs responded; five contributed at four months; and two responded at eight months. Three HCPs contributed twice each. Fourteen respondents provided a total of 20 responses over the course of the study.

**Table 2:** Characteristics of health care providers (N = 14)

Characteristics	N (%)
Age (years)	
Mean (SD)	43.64 (10.66)
Range	27–61
Gender	
Female	12 (85.7)
Male	2 (14.3)
Education	
Completed post secondary	13 (92.9)
Some post secondary	1 (7.1)
Occupation	
Nurse	8 (57.1)
Health care aide	2 (14.3)
Physician	3 (21.4)
Allied health professional	1 (7.1)
Years in Current Occupation	
< 1	1 (7.1)
1–5	3 (21.4)
6–10	3 (21.4)
11–15	1 (7.1)
16–20	2 (14.3)
21–25	2 (14.3)
> 25	2 (14.3)

### Social support and ease of keeping in touch

In terms of the relationships that provide patients with main social support, children (6) and spouses/partners (6) were most frequently identified followed by friends (4), siblings (3), and parents (1). Ten of the thirteen patient participants completed the MSPSS scale, the mean score was M = 74.5 (SD = 7.63); thirty-seven of the 38 family member participants completed the MSPSS, the mean score was M = 73.6 (SD = 9.76), indicating that both groups judged their social support to be relatively high.

Activities that patients missed most while in hospital included spending time with friends or family (84.62%), learning new things (84.62%), doing quiet activities (e.g., reading, watching TV) (76.92%) and physical activities (69.23%). With regard to how hard it is to keep in touch with family and friends, on a scale of 1, “not hard at all” to 7, “very hard”, the mean score for patients was M = 1.92 (SD = 1.44). While the mean score for family members was M = 5.29 (SD = 1.06), indicating that family members experienced some difficulty keeping in touch.

### Participants’ general experience/interest in computer and internet use

In terms of the familiarity with ‘using computers’ and ‘using the internet’, on a scale of 1 “not at all” to 7 “expert”, the mean score for patients was M = 3.67 (SD = 1.07) and M = 3.92 (SD = 1.56) respectively; for family members, M = 5.29 (SD = 1.06) and M = 5.59 (SD = 1.07) respectively. Regarding their interest in using a computer with internet access if it was available at the patient’s bedside, on a scale of 1 “not at all” to 7 “very interested”, the mean scores were M = 5.42 (SD = 1.78) for the 12 patient respondents and M = 6.54 (SD = 0.87) for the 37 family respondents.

### Participants’ experience with the KIT technology

#### Daily logs

Individual participants kept logs of their computer/internet activities for 1 to 8 days, mean of 4.63 days. Fifty-one participants contributed a total of 231 days logged.

#### Keeping in touch

“Keeping in touch with someone” was the most frequently logged internet activity, noted in 83.7% of days logged. The people most frequently contacted were family (75% of days logged), followed by friends (66.7%), co-workers (18.6%), and “others” (11.5%). Locations where people were contacted included Winnipeg (noted in 63.5% of days logged), somewhere in Manitoba other than Winnipeg (21.2%), followed by somewhere else in Canada (53.6%); North America (29.5%), Europe (14.8%), South America (5.6%), Australia/New Zealand (3.9%), Africa (3.23%), and Asia (1.3%). The most frequent means of keeping in touch (indicated in 76.3% of days logged) was email, followed by social networking such as using Facebook (34.6%), live video calls such as Skype (27.1%), live audio calls (6.5%), and “other” (11.6%). While “keeping in touch”, participants most frequently relayed or received information (71.4% of days logged), followed by chatting or visiting (38.7%), accomplishing a task with the person they connected with; (33.6%), keeping up a routine (31.6%), receiving or giving advice or help with a decision (16.1%), “other” (7.7%), and being part of an event (2.6%).

#### Entertainment

On 65.4% of days logged, the computer/internet was used for entertainment. The most commonly reported form of entertainment was surfing the internet (46.0% of days logged), followed by playing a game or doing a puzzle (43.5%), reading a newspaper, book, or magazine (27.6%), “other” (17.2%), watching a TV program or movie (12.0%), listening to music (11.1%), online shopping (5.6%), or listening to a talk (2.3%).

#### Searching information and accomplishing a task

Data indicated that the computer/internet was used to look up information in 54.7% of days logged. In only 28.1% of those days, either patients or family members looked up information about the patient’s illness or care. In the majority of instances, respondents indicated that they looked up “other” information. In addition, patients and families used the computer/internet to accomplish a task (e.g., online banking) in 47.7% of days logged; however, they were not asked to detail the types of tasks.

#### Remote monitoring

**Fig. 1:**
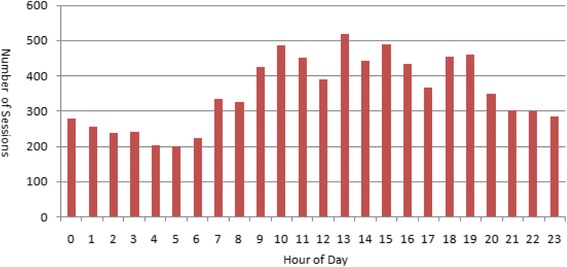
Total number of internet “sessions” by time of day

The hard-copy logs were supplemented by data gathered via the remote monitor. Continuous remote monitoring was used to capture all internet visits to websites. To ensure that this monitored only intentional activity (not simply a connection being inadvertently left open), we define a ‘session’ as activity book-ended by 20 min or more of inactivity. A session could vary in length and have many periods of inactivity, but as long as those periods of inactivity were shorter than 20 min, it was counted as one session. Figure 1 illustrates the total number of internet sessions on any given hour of the day. Participants were most active on the internet from about 10 a.m. to early evening, but activity indeed occurred at all hours, including through the night. Session length ranged between 15 and 25 min, regardless of time of day.

Remote monitoring of all internet activities demonstrated that the greatest proportion of activity on the internet was in the “entertainment” category (78%), which included activities such as: shopping, playing games, viewing news websites, reading on-line magazines, visiting weather websites, reading blogs, viewing videos, or listening to audio feeds. The second most frequent activity, representing 17% of internet activity, included what we called “Keep in Touch” activities: social networking, emailing, chatting, or live video/audio calling. Finally, 5% of the activity was classified as accomplishing tasks such as: on-line banking and bill paying, accessing the Yellow Pages, accessing calendars, or accessing work via virtual private networks.

### Participants’ feelings associated with keeping in touch and daily use of computer/internet

Participants who completed a daily log and indicated that they had kept in touch with someone were asked to rate how keeping in touch and how using the computer/internet had left them feeling (see Tables 3 and 4); 54 to 89% of patients and 84 to 89% of family reported the web-based encounter left them feeling more connected, better, closer and calmer; and between 47 to 69% patients and 64 to 76% family, more relaxed, enhanced well-being, satisfied and more able. Twenty-one family members provided comments about how they felt each day. Some themes in their remarks and examples are shown in Table 5. Nine patients chose to add comments; several commented on multiple days. Generally, patients’ comments were positive, but collecting their feelings over several days sometimes captured the highs and lows of using the technology. For instance, on one day, one patient remarked “I felt connected to my world which is very important when you are in hospital” and, on another day, the same patient said, “I got disconnected on one of my telephone calls. Frustrating!” [PT009]. In another instance, one patient’s comments were quite consistent over several days:

**Table 3:** How keeping in touch and using the computer left patients feeling

Overall, keeping in touch with someone today left me feeling (%):^a^						
	1	2	3	4	5	
Alone	0.0	0.0	10.3	27.6	62.1	Connected
Worse	0.0	7.1	14.3	14.3	64.3	Better
More distant	6.7	0.0	13.3	20.0	60.0	Closer
Agitated	0.0	10.7	35.7	17.9	35.7	Calm
Overall, using the computer today left me feeling (%):^b^						
	1	2	3	4	5	
Tense	3.0	6.1	21.2	33.3	36.4	Relaxed
Diminished well-being	3.1	6.3	43.8	15.6	31.3	Enhanced well-being
Unsatisfied	3.0	3.0	33.3	21.2	39.4	Satisfied
Less able	2.9	0.0	41.2	26.5	29.4	More able

^a^Representing 28 to 30 days logged provided by 13 patients (results of all scales completed within log reports)

^b^Representing 32 to 34 days logged provided by 13 patients (results of all scales completed within log reports)

**Table 4:** How keeping in touch and using the computer left family feeling

Overall, keeping in touch with someone today left me feeling (%):^a^						
	1	2	3	4	5	
Alone	0.0	1.7	9.2	15.0	74.2	Connected
Worse	0.8	5.1	7.6	19.5	66.9	Better
More distant	0.0	5.9	8.5	25.4	60.2	Closer
Agitated	0.8	0.8	14.4	32.2	51.7	Calm
Overall, using the computer today left me feeling (%):^b^						
	1	2	3	4	5	
Tense	0.7	1.4	22.0	31.9	44.0	Relaxed
Diminished well-being	0.0	12.3	23.2	26.8	37.7	Enhanced well-being
Unsatisfied	0.7	11.3	19.8	23.4	44.7	Satisfied
Less able	0.7	10.1	19.4	25.9	43.9	More able

^a^Representing 118 to 120 days logged provided by 38 family members (results of all scales completed within log reports)

^b^Representing 138 to 141 days logged provided by 38 family members (results of all scales completed within log reports)

**Table 5:** Themes and examples of family members’ feeling about keeping in touch (*N* = 21)

*Feeling supported*	
“It left me feeling supported because as soon as my brother received my email about our father, he phoned.” [FM034] “Gave me a sense of relief being able to communicate with family that is not able to be with us at this sad time.” [FM043]	
*Feeling able to accomplish tasks*	
“…having internet access allows me to work from my mother’s room.” [FM008] “Able to accomplish tasks that I won’t have to do when I get home.” [FM034]	
*Feeling more in control of life*	
“I was able to understand and go over information with health professionals over the internet.” [FM036] “It helped me clarify my appointments for the week without leaving the bedside. I felt reassured.” [FM048]	
*Feeling good about being connected to others*	
“Nice to be able to have family close using Skype.” [FM029] “Granddaughter showed us her carved pumpkins and she showed us the grass and trees outside her apartment and the sunny weather outside in Louisiana. It’s rainy here. Keep in Touch made my day happy!” [FM017]

- Day 1 “It is very refreshing being able to be in contact with friends. I feel more in touch and back toward normal”;
- Day 2 “Helped bring things back to the way things used to be”;
- Day 3 “I felt very connected to my old life”. [PT052].

### Participants’ general assessment of their experience with internet-enabled communication

Participants were asked to generally assess in what ways the KIT technology helped them maintain a sense of belonging to family or other social networks, keep up with usual interests, be part of a special event, or whether the technology introduced any new stresses or strains for them. They were also asked to endorse a list of activities associated with their social support networks. The influence of having the bedside KIT technology on patients’ general well-being, QOL, satisfaction with QOL and loneliness were also assessed.

#### Maintain sense of belonging to family or other social networks

Responses to this question were grouped into five major categories: 1) updating the patient’s condition: e.g., “I was able to send my siblings emails detailing our father’s condition” [FM034]; 2) crossing the distance divide: e.g., “It kept me in touch with friends in Texas and my nephew in England” [PT011]; 3) saying a final goodbye: e.g., “Talking to family in other places USA, Newfoundland so we could let them be part of a final goodbye” [FM012]; 4) social support: e.g., “It helped us feel not alone and have some unity with our family” [FM036]; and 5) logistics: e.g., “Kept in touch with kids—where I am, what needed to be done, what the plans for them were” [FM051].

#### Keep up with usual interests

The activities family members performed using the KIT technology to help them keep up with usual interests were synthesized into seven categories: 1) work/business, 2) volunteer work, 3) personal business, 4) entertainment, 5) communication, 6) personal reflection, and 7) information gathering. Patients’ responses to this question mirrored those of family members except that patients did not conduct work or volunteer activities. In a similar vein, patients and family members were asked in what ways the computer and internet helped them accomplish things that they otherwise may not have done while in hospital. Their replies echoed family response categories. Family members listed tasks such as: work, volunteer activities, scheduling, paying bills, banking, continuing regular routines, and coordinating family activities. Patients mentioned tasks such as: paying bills, banking, keeping up with current events, learning to use an iPad, and keeping occupied.

#### Being part of a special event

Patients and family members stated that having internet access in the hospital could allow them to be part of a special event in which they otherwise would not have been able to take part, such as a wedding, a birthday, children’s activities, or simply visiting.

#### New stresses or strains

Most patients and family members stated that there were no new stresses associated with the computer. As with patients, a few family members commented that, contrary to creating strain, the technology reduced it. For example, one respondent commented: “Not stressful at all – it was liberating, if anything.” [FM002]. One patient identified a stressor related to the difficulty of using the iPad while lying in bed. Several family members identified stressors such as the Skype ring-tone annoying the patient, computer cluttering the over-bed table, unreliable internet connection, not fully knowing how to use the technology.

#### Technology-enabled activities

Table 6 lists the technology-enabled ‘keep in touch’ activities endorsed by family members and patients. All patients (100%) reported that KIT helped them connect with people who are important to them and feel back to normal. Nearly all family members (93%) indicated that they used KIT to keep family or friends informed. In addition, a high proportion of both groups (over 70%) agreed that KIT helped them share important thoughts or feelings, and remain part of the lives of significant others.

**Table 6:** Technology-enabled keep in touch activities endorsed by family members (*N* = 28) and patients (*N* = 7)

Using the computer with internet access helped me^a^:	Family members N (%)	Patient N (%)
Connect with someone who is important to me	23 (82.1)	7 (100.0)
Share important thoughts or feelings with someone	23 (82.1)	5 (71.4)
Remain part of the lives of people who are close to me	24 (85.7)	6 (85.7)
Feel part of normal life	22 (78.6)	7 (100.0)
Keep family or friends informed	26 (92.9)	5 (71.4)
Involve family and/or friends in discussions about my (or my family member’s) care	17 (60.7)	1 (14.3)
Be part of events or moments that are important to me	16 (57.1)	3 (42.9)
Other	8 (28.6)	1 (14.3)

^a^Participants could endorse more than one item

#### Self-assessed influence on well-being, QOL and loneliness

Participant were asked whether or not having the bedside KIT technology had a positive influence on their own general well-being, on a scale from 1, “no, not at all” to 5, “yes, very much”. Seven patients responded and the mean score was M = 3.86 (SD = 1.21). Twenty-eight family members responded, and their mean score was M = 4.64 (SD = 0.68). Respondents were asked to rate their quality of life, satisfaction with quality of life and loneliness, before and after KIT. There were non-significant improvements across each of these measure, with the exception of improvement in patient satisfaction in quality of life (*p* = .028) (see Table 7).

**Table 7:** Mean differences for patients (*N* = 6) before and after using KIT

	Before using the KIT technology		After using the KIT technology			
	Mean	(SD)	Mean	(SD)	t	*p*
QOL	4.50	2.43	5.67	3.61	−1.78	.135
Satisfaction with QOL	3.67	2.66	5.50	2.66	−3.05	.028
UCLA Loneliness	37.2	3.90	33.2	10.08	1.36	.247

### Participant—technology interface

More family members than patients (93% versus 71.4%) agreed that they would like to use the computer frequently; likewise, more family members compared to patients (85.7% versus 57.2%) agreed that the computer was easy to use. Family members were more likely than patients (48.1% versus 28.6%) to imagine that people would learn to use the computer very quickly; and a higher proportion of family members than patients (84.6% versus 71.5%) agreed that they felt very confident using the computer. On the other hand, a greater proportion of patients than family members (42.9% versus 10.7%) anticipated that they would need the support of a technical person to be able to use the computer.

#### Participants’ daily assessment of technical quality

Each day as participants completed their daily logs, they were asked to assess several elements of the technical quality of their computer/internet experience that day, on a five-point rating scale (see Tables 8 and 9). Generally, participants found it easy to use the computer and easy to connect to the internet, although there was a small proportion who had a difficult time either using the computer and/or connecting to the internet, or both. Of the participants who used the internet to complete audio/video calls, most rated the sound and picture quality to be excellent. Likewise screen images and website loading speed were favourably judged.

**Table 8:** Participants’ assessment of computer ease of use and internet connection

	How easy/hard was it to use the computer? (% of days logged)					
	Hard				Easy	Did not use
	1	2	3	4	5	6
Patients^a^	9.1	12.1	27.3	6.1	39.4	6.1
Family^b^	3.5	0.0	5.6	12.7	76.1	2.1
	How easy/hard was it to connect to the internet? (% of days logged)					
	Hard				Easy	Did not use
	1	2	3	4	5	6
Patients^c^	9.1	6.1	0.0	6.1	69.7	9.1
Family^d^	2.8	1.4	4.3	7.1	83.7	0.7

^a^Representing 33 days logged provided by 13 patients

^b^Representing 142 days logged provided by 38 family members

^c^Representing 33 days logged provided by 13 patients

^d^Representing 141 days logged provided by 38 family members

**Table 9:** Participants’ assessment of sound, image and loading speed

Technical Element		Quality Rating (% of days logged)^a^					
		Poor-1	2	3	4	Excellent-5	Didn’t use-6
Sound during calls	Patients	0.0	2.9	5.7	8.6	25.7	57.1
	Family	4.3	2.1	2.8	10.6	34.0	46.1
Picture during calls	Patients	3.0	0.0	3.0	9.1	18.2	66.7
	Family	2.8	2.1	4.3	8.5	35.5	46.1
Screen images	Patients	0.0	0.0	14.3	22.9	42.9	20.0
	Family	0.0	0.0	4.2	23.2	64.8	7.7
Website loading speed	Patients	3.0	0.0	3.0	39.4	39.4	15.2
	Family	0.7	0.7	14.4	30.2	48.2	5.8

^a^Representing 33 to 35 days logged provided by 13 patients; and 139 to 141 days logged provided by 38 family members

#### Participants’ technology usability feedback

At the conclusion of the study, participants were asked to rate their agreement with statements related to how easy or hard the computer was to use (see Table 10). The vast majority reported favorable experiences and strong interest in using the computer regularly. Generally, a greater proportion of family members than patients were more likely to learn and use the computer, found the computer easy to use, were more confident using it and less likely to ask for technical support.

**Table 10:** Family members (*N* = 28) and patients (*N* = 7) assessment of ease of using the computer

Indicators of Technology Ease of Use	Patients	Family
	% Agreed	% Agreed
I think I would like to use the computer frequently.	71.4	93.1
I found the computer unnecessarily complex.	0.0	3.6
I thought the computer was easy to use.	57.2	85.7
I think I would need the support of a technical person to be able to use the computer.	42.9	10.7
I would imagine that most people would learn to use the computer very quickly.	28.6	48.1
I found the computer very cumbersome to use.	0.0	14.8
I felt very confident using the computer technology.	71.5	84.6
I needed to learn a lot of things before I could get going with the computer.	14.3	11.1

#### Participants’ technical support satisfaction

Patients and family members were asked how many times over the course of their study they required the help of the research assistant to assist with the technology. Of the five patient respondents, four indicated that they needed help from the research assistant about 4 to 5 times, and one needed help 2 times. However, no significant correlations were found between number of times help was requested and PPS score (*p* = .34), and BOMC score (*p* = .12). Of the 29 family member respondents, 10 said that they did not need help, 9 needed help less than 5 times and 4 needed help more than 5 times.

Participants were also asked to gauge their satisfaction with the technical support they received during the course of their participation in the study. A very high level of satisfaction with technical support was evident among both family members and patients (see Table 11). However, family members were marginally more satisfied with technical support in comparison with patients. Suggestions that respondents made for improving technical support included: having technical support available around the clock and on weekends; better assessment and provision of assistive devices to improve the user-technology interface; and slowing down the speed with which information is provided.

**Table 11:** Family members (*N* = 26) and patients’ (*N* = 7) satisfaction with technical support

Indicators of satisfaction with technical support	Patients	Family
	% Agreed	% Agreed
I was satisfied with the help I received from the research assistant.	85.7	100.0
The research assistant solved the problem I had with the computer.	71.4	61.9
The research assistant arrived in a timely manner.	71.4	100.0
The research assistant explained things in a way that was easy for me to understand.	85.7	100.0
The research assistant seemed genuinely interested in helping me.	85.7	100.0
The research assistant seemed rushed.	14.3	4.0
The research assistant was available when I needed help.	71.5	79.2

#### Willingness to pay

Regarding price tolerance for internet access, only 1 family member (4.0%) was willing to pay more than five dollars per day for internet access, 7 (28.0%) family members and 1 (16.7%) patient would be willing to pay $2.50–5.00 per day, 10 (40%) family members and 3 (50%) patients would be willing to pay less than $2.50 per day, and 7 (28%) family members and 2 (33.3%) patients would not be willing to pay for access to the internet at their bedside.

### Facilitation

Over the course of the study, the research assistant tracked a number of activities related to facilitating participants’ use of the communication technology and trouble-shooting technical problems (see Table 12). Twenty-six of the 51 participants (51%) needed more than 5 to 10 min of the facilitator’s time to help with the technology. The amount of time the facilitator spent interacting with study participants ranged from 15 min to 12 h per person, with an average time of 1.6 h per participant. However, no significant associations were found between facilitation time and PPS score (*p* = .61), and BOMC score (*p* = .42). The only technical problem that frequently vexed study participants and research staff was the unstable internet connection. Because this connection was a temporary installation for the study, there may have been times when the capacity was simply overloaded.

**Table 12:** Typical facilitation activities performed with or for users

Orientate/Encourage	• Assess interests/needs • Help with login • Sit with patient while he accesses email; remove distracting pop-ups for him • Drop-in and scheduled visits offering assistance • Orientate family member to iPad buttons, icons, finger sweeping, pinch
Teach/Demonstrate	• Suggested Google phone to enable and save on long distances costs • Demonstrate how to use iPad or laptop • Orientating user to Skype screen, making a test call, orientating to screen icons during call, and problem-solving how to obtain skype handles for people for whom contact information is not known • Demonstrate use of YouTube to listen to favourite singer • Demonstrate use of front and back camera and where pictures stored • Download games (e.g., Solitaire, Angry Birds) and show user how to play • Teach how to use “home” button • Provide general knowledge of how Facebook works • Taught how to send and receive email
Help	• Bookmark favourite websites • Install free Apps and software customized to users’ hobbies or interests (e.g., apps to run MIDI piano keyboard from iPad) • Sign in to email and other accounts • Set up email link on desktop with automatic login • Physically assist patient to move laptop to overbed table, login, and go to eBay
Set-up/Maintain	• Install standard Apps and software • Exchange different types of equipment according to user needs (e.g., exchange wireless for wired mouse) • Computer “cleaning” to recharge, disinfect and delete all personal information, photos, and browsing history • Work with OT to find appropriate supports for iPad to accommodate patient’s physical constraints • Download users’ pictures to USB for safekeeping • Customized font and display for easier reading • Prepare and test equipment to help family bring a wedding into patient’s room via Skype • Set background on desktop to match user interests (e.g., miniature schnauzers) • Create new accounts (e.g., Skype, Gmail, Facetime) • Update apps and software

### Health care providers’ assessment of advantages and disadvantages of the keep in touch technology

#### Benefits and drawbacks

Benefits observed by health care providers of having computers available at the bedside included: keeping others informed of the patient’s condition, sharing the emotional burden of making important decisions, enjoyment, distraction, visiting around the world, feeling connected to the outside world, able to conduct business and reminiscing. For example:

“Another patient had Skype on and they were seeing their loved ones back home [Eastern Europe]. They were picking mushrooms and laughing so my patient and spouse were laughing, too. Also, remembering when they used to do the same thing back home.” [713B].

Of the 14 health care provider respondents, seven observed no drawbacks associated with the KIT technology. A few drawbacks were identified, including safety concern regarding power cords and “clutter” in the room, difficulty maintaining patient’s undivided attention when the patient was fully engaged with the technology, patients having difficulty using the technology related to age, physical disability, or illness-related confusion.

#### Health care provider recommendations

Health care providers recommended that computers with internet access be provided to patients or family members wanting them. They viewed the technology as an important tool for helping patients and families communicate and stay connected to their family and friends; as a part of daily living; and as a means of enhancing patients’ and family members’ enjoyment and quality of life. Only one individual was reticent about recommending the technology, citing concerns of possible abuse. In terms of improvements, respondents recommended making computers and/or Wi-Fi available as a standard feature on the ward as soon as possible and for everyone; staff being knowledgeable enough to help patients and families with the technology; offering modifications to suit the limitations and capabilities of individuals (such as visual aids); and integrating the technology so that users could use the televisions with computers and internet.

## Discussion

This study explored the feasibility of offering internet-based communication technologies to palliative in-patients and their families and described their experiences with using the technology. The successful application of the KIT technology in palliative care needs to match the interests and needs of patients and their family members. Our results show that most patients and family are interested in using KIT technology. There were relatively few days when the computer/internet was not used (39 of the 231; 16.9% of days logged). Reasons for not using the technology included: not feeling well enough, not having enough time, not being interested, computer not working well, or other. Given the relatively few days participants did not use the computers, not feeling well or issues of time were not barriers to using the technology.

A meaningful dying experience for patients requires the involvement of family and loved ones, regardless of their physical presence [20]. We found that the KIT technology made it easy for patients and families to keep in touch with relatives and friends in various ways such as email, social networking, video and audio calls; it allowed for additional ‘visits’ from family and friends that were otherwise too expensive or time consuming, and it helped patients to accomplish tasks that they might not have undertaken while in hospital. KIT did not replace human contact, but rather provided additional opportunities that otherwise would not be available [27]. Research examining quality of end-of-life care from the perspectives of patients has highlighted the importance of achieving a sense of spiritual peace and maintaining and strengthening relationships with loved ones, with the end of life as a time for renewed intimacy, reconciliation, and life closure [1]. Findings of this study demonstrated the high value that patients and family members place on staying in touch with their social networks and other aspects of their daily lives via KIT technology, making them feel more connected, calm, and supported. Consistent with previous findings [9, 10, 22], patients and family in this study also benefited from access to internet-based information and entertainment, which reduced patient boredom, increased feelings of relaxation, and promoted their satisfaction with quality of life. Unlike previous studies reporting nearly half of patients with cancer use the internet to search for medical information [28], the majority of patients and family members in this study looked up other information instead of information related to illness or care.

An important matter to consider when providing communication devices to patients is how easy the devices are to use. By and large, a greater proportion of family members than patients found the computer easy to use, were more confident using it, and wanted to use it frequently. Whether this finding reflects age differences, generational differences in adaptation to newer communication technologies, or the patients’ illness-related impairments or vulnerabilities was not explored in this study. Most participants were satisfied with the technical quality of the computer, such as ease of use, sound and picture quality. A small portion needed the assistance of a facilitator to make more optimal use of the computer, and patients were more likely to benefit from technical support compared with family members. In this study, participants valued not only knowledgeable and competent technical support but also personal qualities such as friendliness, patience, and a respectful, courteous and caring demeanour.

**Fig. 2:**
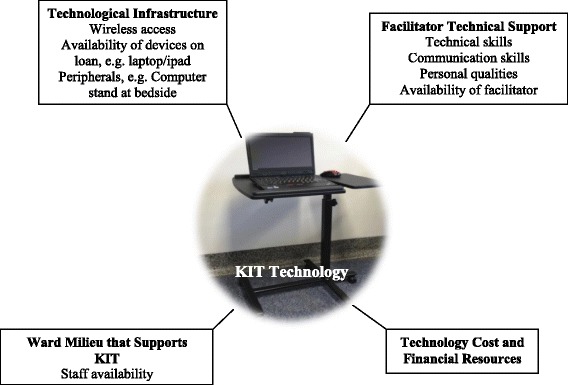
An overarching model of implementation of internet-based communication and information technology in in-patient palliative care

This study revealed that facilitating communication using internet-based technologies for palliative in-patients is a valuable tool, which can be employed with relative ease. Our results provide empirical evidence to support the installation of free or low cost Wi-Fi internet service in in-patient palliative care settings. Based on our findings, an overarching model of implementation of internet-based communication and information technology was developed (Fig. 2). It describes factors that need to be taken into account for successful implementation of internet-based technologies in the in-patient palliative care setting. Our findings can contribute to the design and evaluation of KIT technology in in-patient palliative care. If applicable, facilities may consider providing devices and peripherals on loan to patients and family members, and offer appropriate support services by well-trained facilitators who are proficient with a range of communication and computing devices and possess basic knowledge and skills to support internet use; this is key to successful implementation of technologies [29]. Considering patients would need more technical support, facilitators should be easily accessible to patients, especially during late morning and early evening, which was identified in this study as the peak time for KIT. In addition, factors that identified in the model can potentially inform technology use in palliative care in other settings. However, as Kuziemsky et al. [30] suggested, determinant factors of the successful implementation of technology are context-specific, thus we argue that a thorough context-specific assessment will be needed before implementing the technology.

As telemedicine continues to play an ever-increasing role in palliative medicine, we hope that recognition of the value of KIT technology in patient-to-family communication will grow and its use will expand. Given that this study trialed the use of internet-based technology on an in-patient palliative care unit in a single health centre in Winnipeg, the findings may have limited generalizability. Therefore, the effect of offering internet-based technologies needs to be further explored with a larger sample on palliative in-patient units in diverse locations and in other palliative care settings.

## Conclusions

This study provides empirical evidence to support the provision of internet access throughout the palliative care in-patient unit including an assemblage of devices and personnel to provide technical support. Patients and family members used KIT technology to communicate with friends and family all over the world and in a variety of formats, which made them feel better, connected, closer, and calmer. Healthcare providers recommended that computers with internet access be provided to any patient or family member on the unit who wanted it. Healthcare organizations can enhance the ease with which patients keep in touch with their loved ones and otherwise remain engaged with the world by providing free or low cost wireless internet access and, in some instances, the devices and technical support needed for such access.

## Abbreviations

BOMC: Blessed orientation-memory-concentration
DSL: Digital subscriber line
HCPs: Health care providers
IT: Information technology
KIT: Keep in touch
MSPSS: Multidimensional scale of perceived social support
PPS: Palliative performance scale
QOL: Quality of life
